# Finding Mycenaeans in Minoan Crete? Isotope and DNA analysis of human mobility in Bronze Age Crete

**DOI:** 10.1371/journal.pone.0272144

**Published:** 2022-08-10

**Authors:** Michael Richards, Colin Smith, Olaf Nehlich, Vaughan Grimes, Darlene Weston, Alissa Mittnik, Johannes Krause, Keith Dobney, Yannis Tzedakis, Holley Martlew

**Affiliations:** 1 Department of Archaeology, Simon Fraser University, Burnaby, BC, Canada; 2 Department of Human Evolution, Max Planck Institute for Evolutionary Anthropology, Leipzig, Germany; 3 Laboratorio de Evolución Humana, Universidad de Burgos, Burgos, Spain; 4 Department Archaeology and History, La Trobe University, Melbourne, VIC, Australia; 5 Department of Archaeology, Memorial University of Newfoundland, St. John’s, Newfoundland, Canada; 6 Department of Earth Sciences, Memorial University of Newfoundland, St. John’s, Newfoundland, Canada; 7 Department of Anthropology, University of British Columbia, Vancouver, BC, Canada; 8 Department for Human Evolutionary Biology, Harvard University, Cambridge, MA, United States of America; 9 Department of Genetics, Harvard Medical School, Boston, MA, United States of America; 10 Max Planck−Harvard Research Center for the Archaeoscience of the Ancient Mediterranean (MHAAM), Leipzig, Germany & Cambridge, MA, United States of America; 11 Department of Archaeogenetics, Max Planck Institute for Evolutionary Anthropology, Leipzig, Germany; 12 School of School of Philosophical and Historical Inquiry, University of Sydney, Sydney, Australia; 13 Greek Archaeological Service, Ministry of Culture, Athens, Greece; 14 The Hellenic Archaeological Research Foundation, Cheltenham, United Kingdom; The Cyprus Institute, CYPRUS

## Abstract

We undertook a large-scale study of Neolithic and Bronze Age human mobility on Crete using biomolecular methods (isotope analysis, DNA), with a particular focus on sites dating to the Late Bronze Age (‘Late Minoan’) period. We measured the strontium and sulphur isotope values of animal remains from archaeological sites around the island of Crete to determine the local baseline values. We then measured the strontium and sulphur values of humans from Late Neolithic and Bronze Age sites. Our results indicate that most of the humans have sulphur and strontium isotope values consistent with being local to Crete, showing no evidence for a wide-scale movement of people from the Greek mainland or other areas away from Crete in these time periods. However, we found four individuals from the late Bronze Age (Late Minoan III) cemetery of Armenoi with sulphur isotope values not typically found in Crete and are instead consistent with an origin elsewhere. This cemetery at Armenoi also has one of only a few examples of the newly adopted Mycenaean Linear B script on Crete found outside of the palace sites, pointing to an influence (trade and possible migration) from the mainland, which may then be the place of origin of these four individuals. DNA (mtDNA) studies of eight Late Bronze Age individuals from Armenoi have results consistent with people living in Aegean region at this time and cannot be used to distinguish between individuals from Crete (‘Minoans’) and the Greek mainland [‘Mycenaeans’]).

## Introduction

Archaeological research of the Neolithic and Bronze Age periods on Crete has a long history, starting in the 19^th^ century, and remains a vibrant research area today [1–3]. It is likely that Crete was first substantially settled in the Neolithic period [4–6], with Neolithic levels (especially Late Neolithic) being found at palace sites such as Knossos [7], where the earliest Neolithic levels have recently been radiocarbon dated [8]. There has been substantial research on the Late Neolithic-Early Bronze Age transition in Crete, especially in looking for the origins of what would be a unique later Bronze Age (Minoan) material culture, including the first use of a novel pottery firing technology [9].

The site of Knossos has a long sequence of occupation levels, spanning the early (aceramic) Neolithic, final Neolithic and Early, Middle and Late Bronze Age [10]. The separation into different time periods at the site by Arthur Evans led to the widely use terminology for the chronological sequences on Crete, specifically the use of the term ‘Minoan’ for the Bronze Age levels which is divided into Early, Middle and Late Minoan periods, largely based on pottery typologies [11]. An alternative terminology focusses more on the sequences of the formation and abandonment of the palace sites on Crete and is termed the pre-,proto-,neo- and post-palatial periods [11]. Here, we generally follow the site chronologies used by the site excavators, which use the Evans ‘Minoan’ terminology.

The later Bronze Age, usually labelled the Late Minoan period (corresponding to the final Neopalatial and Postpalatial periods) is an area of particularly intensive study and interest [12, 13]. One of the long-standing areas of interest in Bronze Age Cretan Minoan studies is the end of the period, where many sites were abandoned, and the archaeological record changes to include archaeological material culture and architecture stylistically similar to forms used on the Greek mainland, where the Late Bronze Age is commonly termed ‘Mycenaean’ [13]. This is also the period where there is the appearance of Linear B script in Crete [14], which was widely used in mainland Greece in the Mycenaean period, and the end of the use of the (still untranslated) Linear A writing common in Minoan sites on Crete [15]. The end of the Minoan period is also characterised by the abandonment of palace sites, which often have a ‘destruction layer’ coinciding with the end of the use of these sites. The search for causal factors leading to the end of the Minoan period have been discussed at length since the early days of archaeological studies in Crete [16]. There is an argument that there was a natural disaster, such as an earthquake or volcano, that coincided with this time period, while others have argued that Crete (and the surrounding region) was perhaps violently invaded at this time by external invaders, including the so-called ‘sea peoples’ [16, 17]. There is also the discussion that the island of Crete was settled by Myceneans from the mainland, also perhaps violently, or as new settlers arriving in a perhaps de-populated Crete [18].

**Fig 1:**
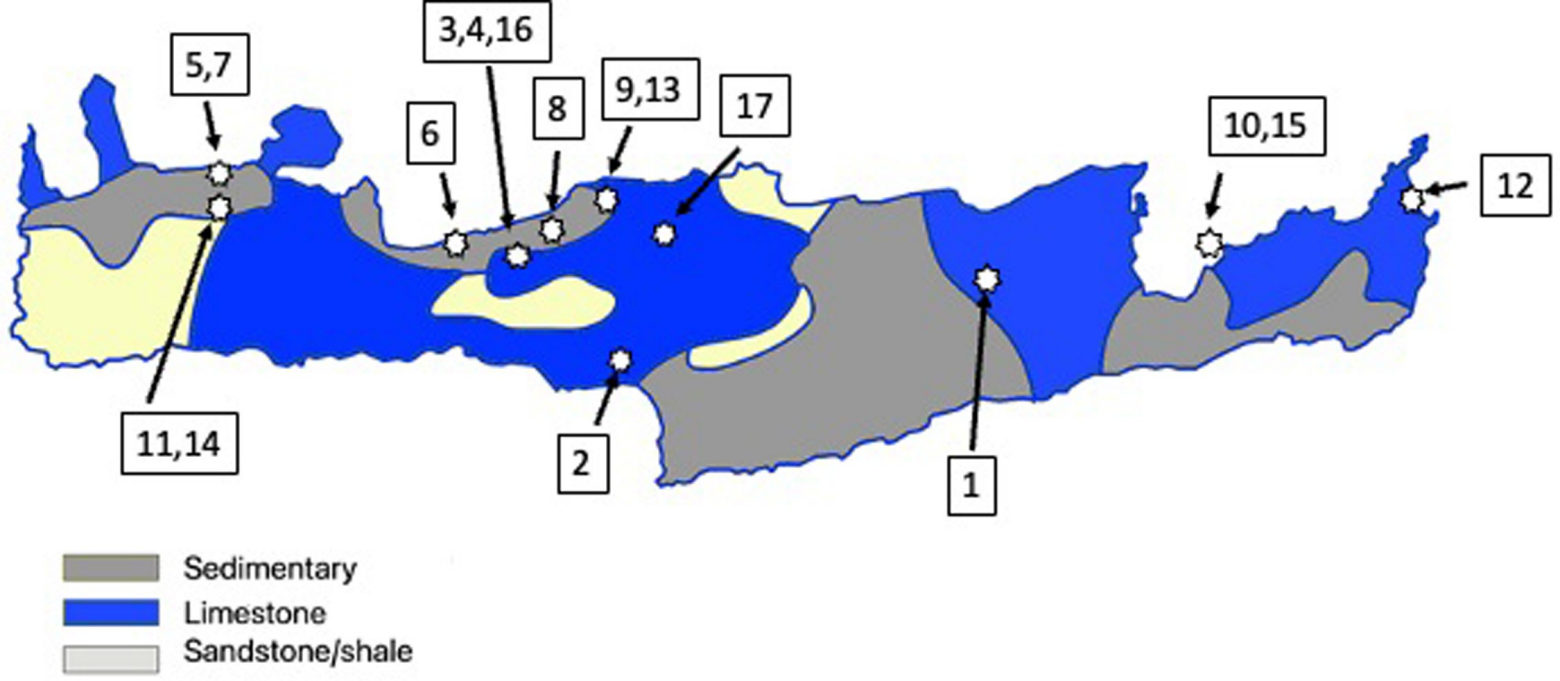
Map of the approximate locations of the study sites in Crete plotted on a simplified geological map (modified after [20]). (1) Agios Charalambos, (2) Apodoulou, (3) Armenoi, (4) Chamalevri, (5) Chania, (6) Gerani, (7) Kastelli, (8) Maroulas, (9) Melidoni, (10) Mochlos, (11) Nerokourou, (12) Palaikastro, (13) Perama Margaritas, (14) Platyvola, (15) Pseira, (16) Vrysinas, (17) Zoniana.

In this paper we sought to explore evidence of mobility in Crete, with an emphasis on sites dating to the Late Minoan periods using biomolecular methods (isotope and DNA analysis). Our study employed a range of isotopic measurements (carbon, nitrogen, strontium, sulphur) of humans and animals from Neolithic and Bronze Age sites across Crete (**Table 1** and **Fig 1**). The isotopic measurements on animals were to establish local baselines for the different regions of the island, and the human isotope values were compared with the local baseline isotope values to see if they were consistent with them living their lives in Crete, and local to the site and region where they were buried. Any humans that had isotope values that were markedly different from the baseline (faunal) values were considered to be ‘non-local’ and most likely from mainland Greece or the Eastern Mediterranean. Mitochondrial DNA was also sequenced from eight individuals and their sequences were compared to larger scale-studies of the region [19] to look for evidence of possible movements of people between Crete and surrounding regions.

**Table 1:** List of study sites and ages of the samples used in this study. Sample Type: H = humans, F = fauna. Age of samples: N = Neolithic, LN = late Neolithic, EM = early Minoan, MM = middle Minoan, LM = late Minoan. Different chronological divisions of these phases are listed using roman numerals (e.g. I, II, III). Site type: C = cave site, N = Necropolis (cemetery), S = settlement. Map number refers to the site location in Fig 1.

Site name	Sample type	Age of samples	Site Type	Map #	Repository
Agios Charalambos	H+F	N to MMIIb	C	1	INSTAP Study Centre for East Crete (Pacheia Ammos, Crete)
Apodoulou	F	MM	S	2	Rethymnon Museum (Rethymnon, Crete)
Armenoi	H+F	LMIII	N	3	Rethymnon Museum (Rethymnon, Crete)
Chamalevri	F	MM to LMIIIC	S	4	Rethymnon Museum (Rethymnon, Crete)
Chania	H	LMIII	N	5	Ministry of Culture Stores, (Chania, Crete)
Gerani	H	LN	C	6	Rethymnon Museum (Rethymnon, Crete)
Kastelli	F	LMIII	S	7	Ministry of Culture Stores, (Chania, Crete)
Maroulas	H	LMIII	N	8	Rethymnon Museum (Rethymnon, Crete)
Melidoni	F	LN	C	9	Rethymnon Museum (Rethymnon, Crete)
Mochlos	H+F	LMI, LMIII	S	10	INSTAP Study Centre for East Crete (Pacheia Ammos, Crete)
Nerokourou	F	MM	S	11	Ministry of Culture Stores, (Chania, Crete)
Palaikastro	H+F	LMIII	S	12	Heraklion Museum (Heraklion, Crete)
Perama Margaritas	H	LMIII	S	13	Ministry of Culture Stores, (Chania, Crete)
Platyvola	H+F	LN	C	14	Ministry of Culture Stores, (Chania, Crete)
Pseira	H+F	LMI, LMIb, LMIII	S	15	INSTAP Study Centre for East Crete (Pacheia Ammos, Crete)
Vrysinas	F	MM	S	16	Rethymnon Museum (Rethymnon, Crete)
Zoniana	F	EM to Roman	C	17	Rethymnon Museum (Rethymnon, Crete)

## Results

### Isotope analysis of faunal remains

**Fig 2:**
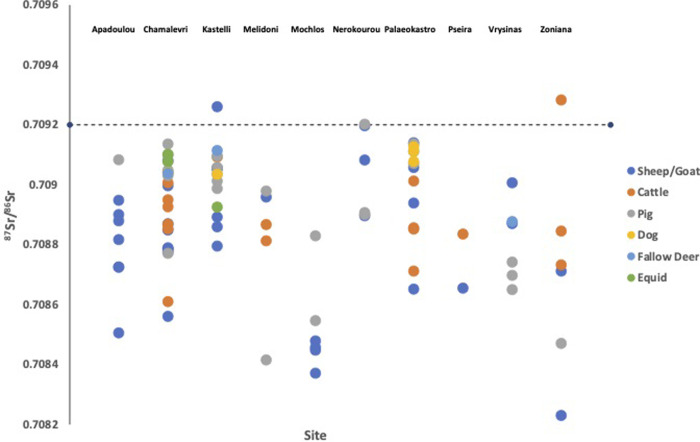
Strontium isotope values of faunal enamel samples from the archaeological study sites in Crete. Also shown is a line indicating the marine strontium isotope value of 0.7092.

**Fig 3:**
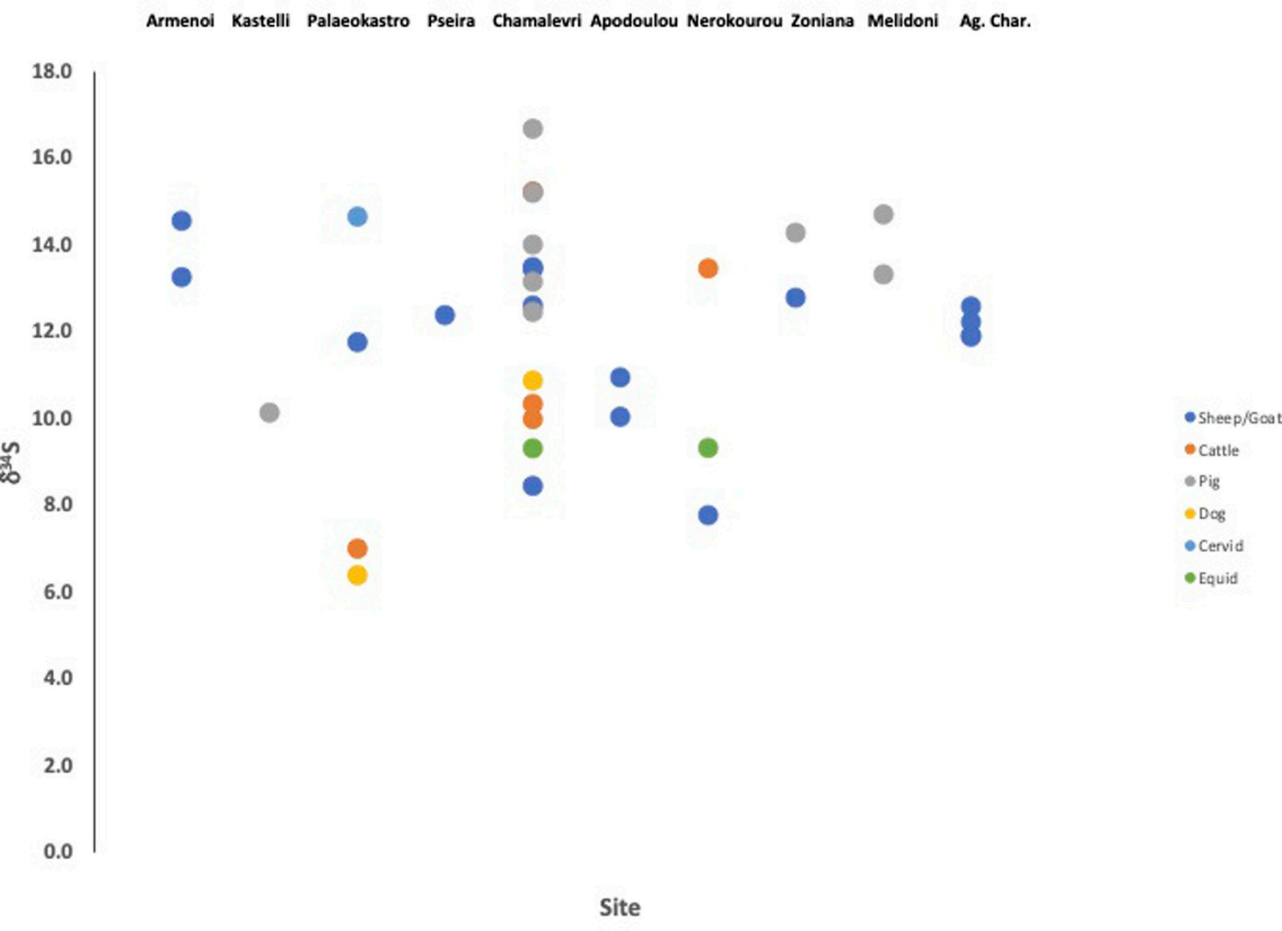
Sulphur isotope values of faunal bone collagen from archaeological study sites in Crete.

We measured the strontium isotope values of 93 zooarchaeological specimens from 10 sites across Crete dating from the Neolithic to Late Bronze Age (**Table 1**). The average faunal enamel strontium isotope (^87^Sr/^86^Sr) value for all samples across Crete was 0.708889 ± 0.000214 (1σ). Additionally, we measured the sulphur isotope (δ^34^S) values of 36 fauna from 10 sites and the average faunal sulphur value is 12.0 ± 2.4‰ (1σ). The results for the strontium isotope measurements of the fauna are plotted in **Fig 2**, and the faunal sulphur isotope values are plotted in **Fig 3**.

The strontium and sulphur isotope values of the animals were very similar across the island, despite there being a variety of underlying geologies (**Fig 1**). We attribute this to the contribution of strontium and sulphur into soils from both sea spray (the ‘sea-spray effect’) and rainfall, which resulted in a more homogenous range of values than we may have predicted. Of note is that the majority of the fauna (90 of 93) and humans (49 of 52) had strontium values lower than the marine strontium isotope ratio of 0.7092 [21]. This indicates that the sea-spray effect where marine strontium was deposited in soils was not significant enough to overwhelm the contributions to soil strontium or sulphur from underlying bedrock (modern-day marine sulphate has a value of δ^34^S = 20.3‰ [22]). And instead most of the bioavailable baseline strontium and sulphur isotope values on Crete are combination of seaspray/rainfall input and the dominant geological substrate, limestones [23].

It is clear in the faunal data (and other data sets [24]) that in general the underlying geologies of Crete impart lower (compared to marine) sulphur and strontium isotope values to fauna on the island and that the sea-spray effect would generate some increases in the values of bioavailable elements. In this context high values (at or above marine) can be considered unusual values.

Three faunal samples have strontium values at, or higher than, the marine strontium average values (**Table 2**). These are from a cattle tooth from Zoniana in central Crete, a pig tooth from Nerokourou and a sheep/goat tooth from Kastelli, which are both sites in West Crete (**Fig 1**). It is possible that these were imported to the island from a region with higher strontium values, such as the Greek mainland or other areas of the Mediterranean, such as Egypt, where we know there was trade with Bronze Age Minoan Crete [25]. It is also possible that these were from a region or regions of Crete with higher strontium values, such as has recently been reported for a region of East Crete [26], which were not sampled in our study.

**Table 2:** The strontium and sulphur isotope values of three faunal samples that have values higher than the marine strontium value and one faunal sample that has a sulphur isotope value outside the average values for fauna from Crete. Also included are the average faunal values for strontium and sulphur isotopes from this study.

Sample	Site	Species	^87^Sr/^86^Sr	δ^34^S
S-EVA 3314 (Tooth)	Zoniana	Cattle	0.709282	n/a
S-EVA 4577 (Tooth)	Nerokourou	Pig	0.709200	n/a
S-EVA 4515 (Tooth)	Kastelli	Sheep/Goat	0.709259	n/a
S-EVA 3260 (Tooth)	Chamalevri	Pig	0.709030	16.7
S-EVA 1192 (Bone)				
Faunal average			**0.708889±0.0002**	**12.2±2.4**
			(n = 93)	(N = 35)

Similarly, the average sulphur isotope values of fauna did not show significant input from higher marine sulphur values (i.e. values at or near to present-day marine sulphate with a value of δ^34^S = 20.3‰ [22]). One pig from the site of Chamalevri in western Crete had a much higher sulphur value than the other fauna, with a value of 16.7‰ (**Table 2**). This value likely indicates that this was in imported animal, although the strontium value for this animal (0.709030) is well within the range of the average (‘local’) faunal values for Crete. And as with the strontium results, it is possible that this pig came from a region of Crete with high sulphur values that we did not sample.

### Isotope results from humans

We measured the strontium values of 52 humans from 9 Neolithic and Bronze Age sites across Crete. We also measured the sulphur isotope values of 72 humans from 4 Neolithic and Bronze Age sites. These results were then compared to the baseline isotope map of Crete developed using strontium and sulphur isotope measurements of animals from these and other archaeological sites across Crete.

**Fig 4:**
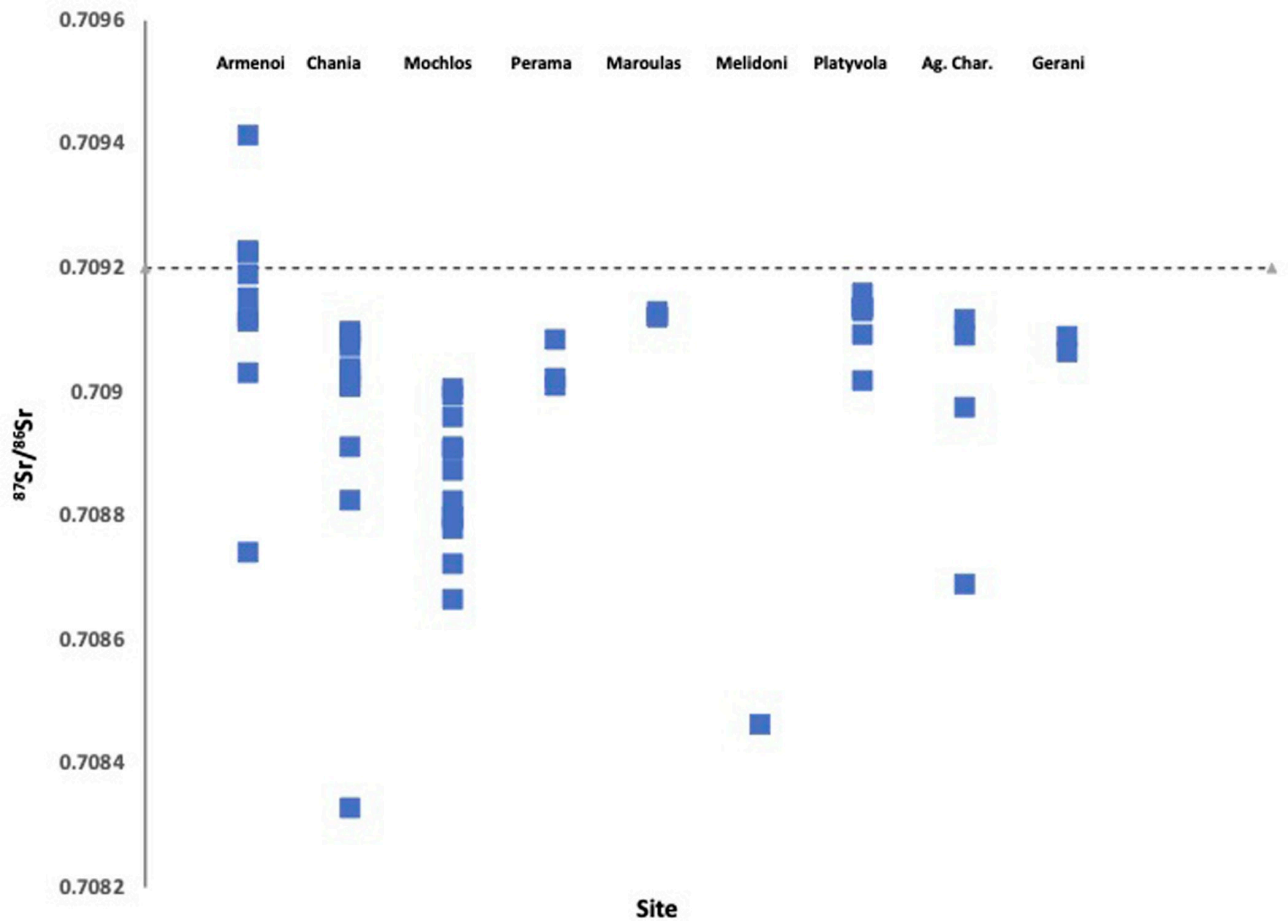
Human strontium isotope values from the archaeological study sites. Also shown is a line indicating the marine strontium value of 0.7092.

**Fig 5:**
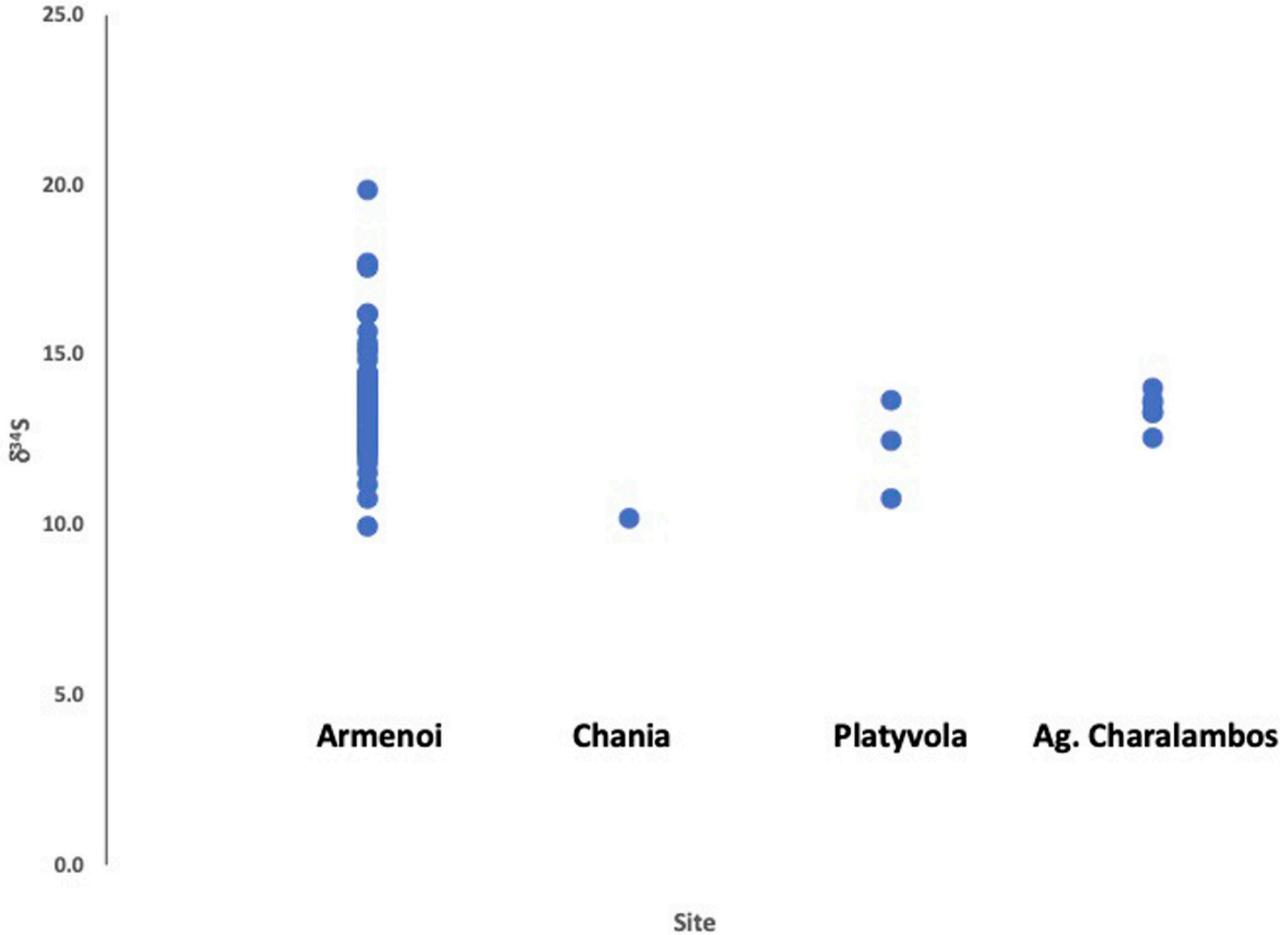
Human bone collagen sulphur isotope values from four study sites.

The sulphur and strontium values of humans from these sites are plotted with the values for sulphur and strontium that we obtained for animals from Crete in **Figs 4 and 5**. The majority of humans had strontium isotope values lower than the marine average strontium value of 0.7092, and most have values that fall within the range of the average strontium value for all fauna from Crete. The exception to this is a number of individuals from a single site (Armenoi) which show values above 0.7092. Likewise, most of these humans also had sulphur values within the average range of the fauna we measured from Crete, but as with the strontium values, some of the individuals from the site of Armenoi had higher sulphur isotope values than the faunal average. We then interpret this result as indicating that the majority of the humans we measured had strontium and sulphur isotope values consistent with their originating from Crete.

The isotope evidence indicates then that the majority of these humans can be considered ‘local’ to Crete. This may indicate a somewhat insular Neolithic and especially Late Minoan society where there was limited migration of people to the Island, and the cemeteries contained mostly local people. This is, of course, is based on the background isotope maps we have produced using faunal enamel strontium values and bone collagen sulphur values, and these baseline values may change with additional future sampling. Another important point is that we have very little data for the strontium and sulphur values from these time periods from the surrounding regions of the eastern Mediterranean, meaning that migrants from regions with similar isotope values to Crete would be invisible as migrants to Crete using these isotope methods.

One important site of note, however, is the Late Minoan (LMIII) cemetery (necropolis) site of Armenoi [19, 27, 28] where four individuals showed sulphur isotope values that were higher (>2 s.d.) than any other measured sulphur values (humans and fauna) from Armenoi itself or other sites across Crete (Table 3). Similarly, two of these same individuals (ARM 501, ARM 504) also showed the highest strontium isotope ratios for humans and higher than all but two of the zooarchaeological samples in our study—higher even than the marine strontium value. Indeed, these two individuals, and a third individual also from Armenoi, are the only humans we measured with strontium values higher than the marine average strontium value. As strontium values record location during childhood tooth enamel formation [29], it could be argued that two of these individuals then may have lived elsewhere than Crete during their childhoods. Sulphur isotope values are from bone collagen, which can reflect dietary intake of sulphur over many years, but specifically in adulthood, so is a useful companion isotope to strontium for mobility studies [22]. Four individuals from Armenoi had sulphur values above 2.s.d from the mean value observed for animals and humans from Crete, they likely lived elsewhere in the years before their death.

**Table 3:** Sulphur, strontium and mtDNA results from four humans with isotope values outside the range of local faunal values for Crete. These individual with ‘non-local’ values are from the Late Bronze Age site or Armenoi, Crete. Also included are the average strontium and sulphur faunal values from this study.

Sample	Sample	Tomb	Sex	Element	^87^Sr/^86^Sr	δ^34^S	mtDNA
S-EVA 3333	501	55 G	M	Tooth	**0.709415**	n/a	T2b25
S-EVA 3519	501	55G	M	Femur	n/a	**17.6**	n/a
S-EVA 3339	504	89 B	M	Tooth	**0.709228**	n/a	HV
S-EVA 3429	504	89 B	M	Femur	n/a	**19.9**	n/a
S-EVA 3450	529	76 D	M	Femur	n/a	**17.6**	n/a
S-EVA 3502	536	95 L	F	Femur	n/a	**17.7**	n/a
Faunal average					**0.708890±0.0002** (n = 93)	**12.2±2.4** (N = 35)	

There was no clear evidence of marine food consumption by these individuals, which may have had an influence on their sulphur or strontium isotope values. The carbon and nitrogen data show a terrestrial-based diet for these humans (**Table 4**), something commonly observed for Bronze Age Greece [30] with the notable exception of Mycenae [31]. The carbon isotope evidence also shows there was no significant consumption of C_4_ foods by humans or animals. It should be noted that the carbon and nitrogen data presented here are from new sample preparations and measurements in Leipzig so the results may differ slightly from the earlier measurements on some of the same samples measured at Oxford [31].

**Table 4:** Table of the sulphur isotope values of humans and fauna from the study sites. Also included are bone collagen carbon and nitrogen isotope values.

Lab # (S-EVA-)	Sample #	Site	Species	δ^34^S	δ^13^C	δ^15^N	C:N	%Collagen	%S
3396	AC-2	Agios Charalambos	Human	13.7	-19.5	8.3	3.4	2.6	0.15
3402	AC-4	Agios Charalambos	Human	12.6	-19.6	9.0	3.2	1.4	0.12
3405	AC-5	Agios Charalambos	Human	13.6	-19.5	8.3	3.2	1.3	0.17
3406	AC-6	Agios Charalambos	Human	13.3	-19.6	9.0	3.2	1.8	0.17
3407	AC-7	Agios Charalambos	Human	14.0	-19.7	9.1	3.2	2.1	0.17
3408	AC-8	Agios Charalambos	Human	13.3	-19.5	8.3	3.3	2.2	0.13
3380	AB-34	Agios Charalambos	Sheep/Goat	11.9	-19.8	6.3	3.1	2.0	0.12
3375	AB-25	Agios Charalambos	Sheep/Goat	11.9	-19.7	8.4	3.1	6.1	0.16
3387	AB-59	Agios Charalambos	Sheep/Goat	12.2	-20.3	6.0	3.2	1.9	0.15
3386	AB-58	Agios Charalambos	Sheep/Goat	12.6	-20.3	4.9	3.2	1.1	0.15
3384	AB-49	Agios Charalambos	Mammal	14.1	-19.9	4.6	3.1	1.4	0.14
1272	Apodoulou-7(27)	Apodoulou	Sheep/Goat	10.0	-19.9	5.3	3.3	1.9	0.15
1277	Apodoulou-12(32)	Apodoulou	Sheep/Goat	10.9	-19.5	5.5	3.3	2.3	0.19
3426	ARM-500	Armenoi	Human	13.6	-20.2	7.8	3.3	2.1	0.21
1263	ARM-503 Tomb 160 (40)	Armenoi	Human	12.8	-20.0	7.8	3.3	1.9	0.18
1264	ARMi-504 Tomb 200 (41)	Armenoi	Human	14.9	-19.7	8.8	3.4	1.0	0.22
3428	ARM-503	Armenoi	Human	13.9	-19.7	8.2	3.3	1.0	0.16
3429	ARM-504	Armenoi	Human	19.8	-19.5	8.3	3.2	1.3	0.17
3430	ARM-505	Armenoi	Human	16.2	-19.7	8.6	3.2	1.5	0.20
3433	ARM-508	Armenoi	Human	14.1	-19.6	8.4	3.2	1.8	0.17
3434	ARM-509	Armenoi	Human	13.9	-19.6	9.4	3.3	2.0	0.22
3435	ARM-510	Armenoi	Human	12.7	-19.4	8.6	3.3	0.9	0.20
3436	ARM-511	Armenoi	Human	13.7	-19.8	8.6	3.4	0.8	0.22
3437	ARM-512	Armenoi	Human	13.1	-19.8	9.2	3.3	3.6	0.20
3439	ARM-514	Armenoi	Human	15.2	-19.6	8.9	3.3	1.5	0.18
3440	ARM-515	Armenoi	Human	16.2	-19.8	8.8	3.3	1.1	0.18
3441	ARM-516	Armenoi	Human	15.4	-19.8	8.6	3.3	1.0	0.19
3445	ARM-520	Armenoi	Human	15.7	-19.6	8.7	3.3	2.2	0.27
3446	ARM-523	Armenoi	Human	13.6	-19.6	8.3	3.3	2.2	0.15
3447	ARM-525	Armenoi	Human	14.3	-19.6	8.7	3.3	1.3	0.21
3448	ARM-526	Armenoi	Human	14.3	-19.7	8.4	3.3	2.2	0.20
3449	ARM-527	Armenoi	Human	11.2	-19.7	9.1	3.4	1.9	0.22
3450	ARM-529	Armenoi	Human	17.6	-19.6	9.1	3.3	1.5	0.21
3451	ARM-530	Armenoi	Human	12.2	-19.6	9.9	3.3	1.7	0.17
3453	ARM-532	Armenoi	Human	10.0	-19.8	8.9	3.4	1.2	0.21
3454	ARM-533	Armenoi	Human	13.3	-19.7	8.0	3.2	1.8	0.16
3455	ARM-535	Armenoi	Human	13.3	-19.7	8.4	3.3	1.4	0.20
3456	ARM-541	Armenoi	Human	14.1	-20.1	8.2	3.2	2.1	0.24
3457	ARM-542	Armenoi	Human	14.0	-20.0	8.3	3.2	1.8	0.19
3458	ARM-543	Armenoi	Human	12.4	-19.6	9.6	3.2	1.8	0.21
3459	ARM-544	Armenoi	Human	12.6	-19.6	8.8	3.3	2.6	0.14
3460	ARM-545	Armenoi	Human	14.5	-19.9	8.9	3.2	2.2	0.20
3461	ARM-546	Armenoi	Human	14.0	-20.3	8.2	3.5	1.5	0.20
3463	ARM-548	Armenoi	Human	14.5	-19.7	8.5	3.2	1.0	0.23
3464	ARM-549	Armenoi	Human	13.5	-19.8	8.5	3.2	1.8	0.18
3465	ARM-550	Armenoi	Human	12.8	-20.3	8.4	3.4	1.5	0.16
3466	ARM-551	Armenoi	Human	13.5	-19.6	8.6	3.3	1.6	0.19
3467	ARM-555	Armenoi	Human	12.3				1.3	0.19
3468	ARM-556	Armenoi	Human	12.5	-20.2	8.5	3.6	1.1	0.15
3469	ARM-557	Armenoi	Human	13.3	-19.7	9.4	3.4	1.3	0.18
3470	ARM-558	Armenoi	Human	11.8	-19.9	8.6	3.3	2.6	0.15
3473	ARM-562	Armenoi	Human	12.4	-19.7	8.0	3.3	1.1	0.17
3474	ARM-563	Armenoi	Human	11.5	-19.8	7.8	3.3	1.3	0.22
3475	ARM-564	Armenoi	Human	12.1	-19.5	8.3	3.3	1.3	0.14
3477	ARM-566	Armenoi	Human	14.1	-19.7	8.3	3.3	1.8	0.24
3478	ARM-567	Armenoi	Human	13.9	-19.8	8.1	3.4	1.3	0.19
3480	ARM-569	Armenoi	Human	14.0	-19.8	8.9	3.3	1.4	0.20
3484	ARM-574	Armenoi	Human	13.5	-19.6	9.0	3.3	1.4	0.22
3485	ARM-575	Armenoi	Human	13.1	-19.6	9.1	3.3	1.7	0.20
3487	ARM-577	Armenoi	Human	12.2	-19.6	9.2	3.3	1.9	0.22
3489	ARM-580	Armenoi	Human	15.1	-19.7	8.4	3.3	1.0	0.26
3490	ARM-581	Armenoi	Human	13.4	-20.0	8.2	3.3	1.1	0.22
3496	ARM-586	Armenoi	Human	10.8	-19.7	9.1	3.2	1.6	0.19
3498	ARM-588	Armenoi	Human	13.5	-19.9	8.1	3.3	2.2	0.20
3499	578/592	Armenoi	Human	12.1	-19.9	8.8	3.2	1.4	0.22
3501	ARM-534	Armenoi	Human	12.8	-19.8	7.9	3.3	1.5	0.17
3502	ARM-536	Armenoi	Human	17.7	-19.8	8.5	3.3	1.1	0.21
3503	ARM-537	Armenoi	Human	13.2	-19.7	8.7	3.3	1.4	0.18
3504	ARM-538	Armenoi	Human	14.4	-19.7	9.1	3.3	1.3	0.20
3507	ARM-552	Armenoi	Human	12.0	-19.7	8.3	3.4	1.1	0.20
3510	ARM-578	Armenoi	Human	12.3	-20.0	8.7	3.3	2.0	0.20
3516	ARM-500(?)	Armenoi	Human	13.9	-19.8	7.7	3.3	2.6	0.18
3519	ARM-501	Armenoi	Human	17.6	-19.4	8.8	3.2	1.0	0.23
3520	ARM-521	Armenoi	Human	13.0	-19.6	9.0	3.2	1.4	0.18
3521	ARM-522	Armenoi	Human	13.8	-19.5	8.8	3.2	1.2	0.21
3511	ARM-654	Armenoi	Sheep	13.3	-19.6	4.8	3.3	1.9	0.15
3512	ARM-655	Armenoi	Sheep	14.6	-20.0	5.5	3.1	2.0	0.18
1198	Chamalevri-5 (47)	Chamalevri	Sheep/Goat	8.4	-19.3	3.9	3.3	2.7	0.18
1202	Chamalevri-14 (56)	Chamalevri	Sheep/Goat	12.6	-19.9	3.9	3.2	1.9	0.16
1212	Chamalevri-22 (64)	Chamalevri	Sheep/Goat	13.5	-17.8	4.7	3.2	3.6	0.16
1196	Chamalevri-12 (54)	Chamalevri	Sheep/Goat	13.5	-19.8	3.3	3.3	1.5	0.14
1201	Chamalevri-11 (53)	Chamalevri	Pig	12.5	-20.3	6.6	3.2	2.6	0.17
1195	Chamalevri-10 (52)	Chamalevri	Pig	13.2	-20.3	5.9	3.2	1.4	0.16
1197	Chamalevri-13 (55)	Chamalevri	Pig	14.0	-20.8	5.0	3.2	1.3	0.18
1200	Chamalevri-7 (49)	Chamalevri	Pig	15.2	-19.5	6.6	3.2	1.3	0.13
1192	Chamalevri-4 (46)	Chamalevri	Pig	16.7	-20.6	4.9	3.2	2.3	0.18
1191	Chamalevri-3 (45)	Chamalevri	Cattle	10.0	-18.8	4.8	3.2	1.7	0.17
1214	Chamalevri-29 (71)	Chamalevri	Cattle	10.3	-20.9	3.7	3.2	1.5	0.16
1217	Chamalevri-28 (70)	Chamalevri	Cattle	15.2	-19.4	6.0	3.3	1.4	0.17
1193	Chamalevri-8 (50)	Chamalevri	Dog	10.9	-19.8	7.4	3.2	4.2	0.18
1208	Chamalevri-20 (62)	Chamalevri	Equid	9.3	-19.2	3.2	3.2	2.0	0.18
4551	Chania-3 T.27	Chania	Human	10.2	-19.7	8.6	3.3	1.9	0.20
4540	Kastelli-20	Kastelli	pig	10.1	-21.3	6.3	3.2	1.6	0.15
1221	Melidoni-3(13)	Melidoni	Pig	13.3	-20.7	6.5	3.2	2.5	0.17
1224	Melidoni-6 (16)	Melidoni	Pig	14.7	-21.1	4.9	3.2	1.6	0.25
4580	Nerokourou-9	Nerokourou	Sheep/Goat	7.8					0.27
4579	Nerokourou-8	Nerokourou	Cattle	13.5					0.15
4578	Nerokourou-7	Nerokourou	Donkey	9.3					0.15
4468	PK-5	Palaikastro	Dog	6.4	-20.4	7.6	3.2	2.8	0.12
4491	PK-21	Palaikastro	Cattle	7.0	-20.3	7.1	3.2	2.3	0.13
4486	PK-17	Palaikastro	Goat	11.8	-19.5	4.9	3.3	1.3	0.17
4500	PK-26	Palaikastro	Cervid	14.6	-19.3	4.4	3.3	1.8	0.18
1282	Platyvola-1	Platyvola	Human	10.8	-19.8	7.8	3.2	0.6	0.14
1285	Platyvola-4	Platyvola	Human	13.7	-19.9	7.8	3.2	1.1	0.20
1291	Platyvola-10	Platyvola	Human	12.5	-19.7	7.7	3.3	1.5	0.16
4604	Pseira-1	Pseira	Sheep	12.4	-20.2	5.2	3.2	7.9	0.16
1237	Zoniana-9 (9)	Zoniana	Sheep	12.8	-20.7	4.0	3.2	3.6	0.16
1234	Zoniana-6 (6)	Zoniana	Pig	14.3	-21.6	4.5	3.2	1.6	0.15

The two individuals with both outlier strontium and sulphur values likely spent their childhood and much of their adult lives away from Crete and perhaps then were recent arrivals in Crete before their death and internment in Armenoi.

### DNA results for humans

The DNA preservation was overall rather poor, with the percentage of endogenous human DNA in every sample between 0.01 and 0.18 (Table 6). Due to the low yield of human shotgun reads, the genetic sex could not be determined. Nevertheless, the mitochondrial capture resulted in 10 complete or nearly complete mtDNA genomes, with reads showing patterns of C-to-T and G-to-A misincorporations at the 5’ and 3’ ends, respectively, which are typical of authentic ancient DNA. We estimate the mitochondrial contamination as 3% or lower for all 10 genomes. The mtDNA evidence shows that most of the people we analysed had sequences typical for this region and time period [19]. For the ten individuals analysed from Late Bronze Age Armenoi and Neolithic Gerani, we did not find any individuals with DNA haplogroups that are unexpected for this region. At Armenoi we likely detect several maternal related individuals, who all carry the identical haplogroup H59.

## Discussion

The biomolecular evidence presented here strongly points to little movement of people between Crete and the surrounding regions during the Neolithic and Bronze Age periods. However, at the end of the Bronze Age, in the postpalatial Late Minoan period (LMIII) we found four individuals with non-local isotope values suggesting they may have originated from outside of Crete and were then buried in the Late Minoan cemetery of Armenoi.

The site of Armenoi dates to the Late Minoan IIIB (LMIIIB) period and contains over 230 burial features. It has been excavated by Y. Tzedakis over a period of more than 40 years, first starting in 1969 and has many rock-cut burial features (chamber tombs) of different sizes [19, 27, 28]. Some of the larger tombs contained grave goods including pottery and burials in large free-standing ceramic *larnakes*. Many of the other tombs also contained pottery, and some of the smaller ones had no grave goods at all. These chamber tombs usually contained either multiple or single burials. Most of the pottery dates to the LMIIIB period, and there is the remarkable find of a pottery vessel (stirrup jar) from tomb 146 (which has DNA results presented in this paper, but we were unable to measure isotope values of humans from this tomb) that is inscribed with Linear B script (the inscription is ‘*wi-na-jo*’). The Linear B writing script was not in use in Early or Middle Bronze Age Crete (which instead used Linear A script), but it was in widespread use at Late Bronze age Mycenaean sites on the Greek mainland. Therefore, Armenoi (and some of the palace sites, such as Knossos, where this new Linear B script is the main script used on tablets in the Late Bronze Age) may have been settled by people from the mainland who used this Mycenaean script or may have been imported from the mainland through trade or other contacts and was then locally adopted as the main script.

It is possible then that the four individuals with ‘non-local’ sulphur isotope values (two of these individuals also had higher strontium isotope values than the marine strontium value and our bioavailable faunal baseline average value) were new Mycenaean settlers or traders (especially the two individuals with both strontium and sulphur outlier values) from the mainland that settled at or near to Armenoi and the existing cemetery was used for burials of these newcomers.

While the mtDNA sequences do not offer conclusive evidence for the origins of the studied individuals, previously published nuclear DNA data from Armenoi specimen 503 showed that this individual was genetically distinct from preceding Cretans, and in her genetic profile more similar to contemporaneous Myceneans from the mainland in that she also harbored ancestry derived from Bronze Age steppe pastoralists that Minoans pre-LMIII lacked [19].

### Previous work

There have been two previous studies of strontium isotopes applied to Bronze Age archaeological sites in Crete. The first, Nafplioti [32] presented strontium data from the site of Knossos. As with our study, most of the humans measured showed enamel strontium values below the marine strontium value of 0.7092. There were, however, a number of exceptions, with a few individuals having values higher than the marine strontium value, and with strontium isotope values that were very similar to the two individuals with these high values from Armenoi reported here. Interestingly, Knossos is the main palace site on Crete where Linear B script has been found in the Late Bronze Age (LMIII) period, and Linear B was also found at Armenoi.

A second, more recent study [26] applied strontium isotopes to the early and middle Bronze Age site of Sissi in east Crete. Here, strontium values of four snails from near to the site were used as being indicative of the ‘local’ strontium value. Three of these modern snail shells showed an average value below 0.7092 and within the range of values we observed for zooarchaeological enamel values from the island (0.70830 +/- 0.00062). The fourth snail shell showed a much higher strontium isotope value of 0.70974, higher than all of the zooarchaeological enamel strontium values we measured in our study across Crete. Interestingly at this site, the human enamel samples almost all had strontium values higher than the marine value of 0.7092, which is much higher than the majority of humans and animal data presented here and previously, as well as in three of the four snails from the site. Is this perhaps one localized region of the island that does have higher strontium values than elsewhere? Clearly, further work is needed to better establish the strontium baselines across Crete at higher resolution. With an increased dataset from Crete perhaps we will be able to provide additional evidence that this area of Crete did have high strontium baseline values in the Bronze Age, and also if there are other areas of Crete that also may have had these higher strontium values at this time. With more strontium baseline values from Crete (especially from the surrounding areas of the eastern Mediterranean) we will be better able to indicate possible locations where the individuals we and others have identified with higher strontium values (greater than the marine value of 0.7092) may have originated.

One study attempted to produce a widescale map of the bioavailable strontium values from across all of Greece, and included data from six sites on Crete [24] (and these results are also included in a more recent paper by Frank et al. [33]) The strontium data from these six sites derive from archaeological bone samples (not tooth enamel), which is not a reliable substrate for strontium measurements as it is often contaminated by post-depositional uptake of strontium from soil [29, 34]. Although these results should be used with caution, which was also acknowledged by Nafplioti [24], the results from these six sites (that include some of the same sites we reported in our study) all show strontium values lower than the marine strontium value and are in line with the strontium values we obtained from animal teeth from the same, or nearby, sites.

## Conclusions

The archaeology of Neolithic and Bronze Age Crete is among the longest and most studied regions and time periods in the world. This is particularly the case for the Late Bronze Age-Late Minoan periods. The end of the Bronze Age and the associated first appearance of Mycenaean material culture and architectural styles on Crete is particularly intriguing. Here we sought to add to the body of evidence for human mobility in these important time periods using biomolecular isotope and DNA methods. Our results point to a largely insular Neolithic and Bronze Age Crete, with little evidence of the movement of people to the island. We did find the exciting possibility of identifying newcomers to Crete at the end of the Minoan period, perhaps from mainland Mycenean Greece, at the LMIII necropolis/cemetery site of Armenoi, a site which also has evidence of Mycenaean Linear B script on a pottery vessel from this site at this time. Of course, this could have been an adoption of the written language and material culture from the mainland, or part of a trade network with Mycenaeans, rather than a movement of people. However, if our isotope evidence does indeed show that there were some individuals buried at Armenoi at this transition period at the end of the Minoan era that did not originate from Crete, we may then have evidence that this new writing style was brought to Crete by people moving from mainland Mycenaean Greece who were then buried at the cemetery in Armenoi.

## Materials and methods

### Study sites

We sampled Neolithic and Bronze faunal and humans for isotope and DNA analysis from across Crete for this study. We sought to sample as many sites as possible across a wide geographic range of Crete. We sampled as many sites as we were able to, depending on the availability and accessibility of the material, and the generous collaborations with our many collaborators and the Greek Ministry of Culture. The list of sites and their chronology is given in **Table 1** and their locations are plotted in **Fig 1**. Samples were taken by MPR, HM and KD, with zooarchaeological identifications by KD. Permits for sample export and analysis for all samples were obtained from the Greek Ministry of Culture. All necessary permits were obtained for the described study, which complied with all relevant regulations.

### Isotope analysis

#### Background

The use of strontium isotope analysis to trace mobility has been widely used in archaeology since the late 1980’s [29, 35]. Strontium in mammal tooth enamel originates from food and (to a lesser extent) water in diets and it substitutes for calcium during tooth enamel formation. The strontium isotope ratio between amounts of two isotopes of strontium, ^87^Sr and ^86^Sr (reported as ^87^Sr/^86^Sr) that were present in food and water are preserved in the enamel when the strontium is deposited, so are directly related to the strontium isotope ratio (^87^Sr/^86^Sr) of food and water that was being consumed during enamel formation. In turn the food (i.e. plants and animals) and water strontium ratios are related to the soils and water environment where the food was grown or raised. Therefore, the strontium isotope ratio in enamel can be used to tell us the geographical source of the food that contained the strontium that was eventually deposited in teeth. To use this strontium isotope method as a mobility indicator for humans, it is essential to understand the ^87^Sr/^86^Sr ratios of the geographical areas that were potential food and water sources. This is usually done by first exploring the geological maps of a region and predicting the strontium isotope values of plants and soils based on the age and type of the underlying bedrocks. As the actual human strontium isotope ratios are not directly related to bedrock geology strontium, where possible a map of the strontium that is actually incorporated into plants and animals (‘bioavailable’ strontium) needs to be produced, often by measuring the strontium of animal remains from different regions, as well as sampling modern plants and molluscs (e.g. [36, 37]). Once the map of strontium isotope ratios in a region is produced, the human ^87^Sr/^86^Sr ratios are compared to the map to determine if the values match the ‘local’ values, or not (‘non-local). With this method then, the best use of it is to determine if humans are local or non-local to a region, and if they are non-local, it is not usually possible to determine definitively their origin as there simply are not enough region-specific detailed bioavailable strontium isotope maps available (however see [38] for a first attempt at a global scale-modelled map), therefore many regions of the world do not have these baseline strontium values based on empirically-derived data. Also, it is important to note that for humans, enamel is usually formed during childhood (depending on the specific tooth) so the strontium values of the enamel of permanent teeth of an adult likely indicates where that person was getting their food from while they were children.

Sulphur isotope analysis, on the other hand, is measured in bone collagen so can be used as an indicator of mobility in later life, depending on which bone was sampled [22]. Similar to strontium, sulphur is derived from diet, however sulphur is deposited in bone collagen in the amino acid methionine [22, 39, 40]. Sulphur is both a dietary and mobility indicator, as it is very useful to determine which ecosystem dietary protein was derived from, and can be used to determine freshwater proteins (e.g. fish) compared to terrestrial based proteins (e.g. terrestrial herbivore meat and milk) [41, 42]. As plant sulphur isotope values are related to the soil sulphur isotope values these ratios in human collagen can, in a very similar way to strontium, be used to determine the location where an individual obtained most of their foods when the collagen was being synthesised and deposited within bones.

The combination of strontium and sulphur isotope analysis is then a powerful tool to help reconstruct the life history of a person, allowing the comparison of childhood location with where they were living in later life.

#### Sample preparation

Samples of tooth and bone were prepared and analysed at the Department of Human Evolution, Max Planck Institute for Evolutionary Anthropology, Leipzig, Germany. Collagen was extracted from bone for carbon, nitrogen and sulphur isotope analysis in 0.5M HCl at 5°C following established protocols outlined in [43] with the addition of an ultrafiltration step [44]. The %C, %N and where measured, %S were determined for each collagen extract and only those with acceptable collagen preservation criteria (% collagen yield, C:N and C:N:S ratios, [45, 46]) were included here, and these values are given in **Table 4**. Carbon and nitrogen isotope measurements were undertaken using a Thermo Flash EA coupled to a Thermo Delta V continuous flow isotope ratio mass spectrometer (CF-IRMS). Sulphur isotope measurements of collagen were undertaken on a Heka EuroVector elemental analyser Thermo-Finnigan Delta V plus following procedures outlined in [40]. Isotope ratios were calculated in relation to international and internal standards. Specifically, methionine (Brad-001) and NBS Liver 1577b internal standards (both externally certified) and the international IAEA N1, N2, CH6 and CH7 standards for carbon and nitrogen measurements. The sulphur measurements were calculated using the international standards NBS127, IAEA S1, S2, S3, SO-5 and NIST1577b. Errors on the isotope measurements are (1σ) δ^13^C ± 0.1‰, δ^15^N ± 0.2‰, and δ^34^S ± 0.5‰, based on long-term measurements of the internal secondary standards and international standards listed above.

The strontium isotope measurements of the faunal and human teeth were made on solutions of purified and isolated strontium prepared from sampled tooth enamel. The preparation protocol is described in [47]. This involved digestion of ~20mg enamel, previously mechanically abraded and ultrasonicated in Deionized (DI) H_2_O (18.3MΩ), in 1ml of double-distilled 14.3M nitric acid (HNO_3_) at 120°C for 1 hour within a closed 3ml PFA vial (Savillex). The resulting solution was dried and the sample residue transferred in 1ml of 3M HNO_3_ to a 2ml chromatography column (Eichrom) containing previously cleaned Sr-spec resin (Eichrom). Following three successive 1ml washes of 3M HNO_3_, strontium was eluted from the column into a clean 3ml PFA vial (Savillex) using 2ml of DI H_2_O. The strontium containing solution was then dried and re-dissolved in 3% HNO_3_ for analysis. The strontium isotope ratios were measured using a Thermo Fisher Neptune MC-ICP-MS with instrument parameters and data collection method outlined in [48]. Each sample ^87^Sr/^86^Sr value is the result of 50 analyses (i.e., 50 cycles at 2 second integrations) and involved interference (from krypton and rubidium) and mass bias (^88^Sr/^86^Sr = 8.375209) normalization [49] and inverse mass bias correction. Within the data collection analytical sessions, the NIST strontium carbonate standard SRM987 was also measured as a 3% HNO_3_ solution at 200ppb Sr and gave ^87^Sr/^86^Sr = 0.710262 (+/- 0.000017 1σ, n = 30). All ^87^Sr/^86^Sr sample data were externally adjusted such that SRM987 ^87^Sr/^86^Sr = 0.710240, resulting in typical data corrections of <0.00004, and total procedural blanks were considered a negligible (e.g. <0.5%) contribution to the sample ^88^Sr signal. The strontium isotope data is given in **Table 5**.

**Table 5:** Table of the strontium isotope data from human and faunal enamel from the study sites.

Lab # (S-EVA-)	Sample #	Site	Species	^87^Sr/^86^Sr
3395	AC-1	Agios Charalambos	Human	0.708690
3398	AC-2	Agios Charalambos	Human	0.709118
3401	AC-3	Agios Charalambos	Human	0.708976
3404	AC-4	Agios Charalambos	Human	0.709092
3345	Apodoulou-6(26)	Apodoulou	Sheep/Goat	0.708505
3342	Apodoulou-3(23)	Apodoulou	Sheep/Goat	0.708725
3344	Apodoulou-5(25)	Apodoulou	Sheep/Goat	0.708725
3351	Apodoulou-16(36)	Apodoulou	Sheep/Goat	0.708816
3350	Apodoulou-15(35)	Apodoulou	Sheep/Goat	0.708879
3340	Apodoulou-2(22)	Apodoulou	Sheep/Goat	0.708900
3349	Apodoulou-13(13)	Apodoulou	Sheep/Goat	0.708948
3348	Apodoulou-11(31)	Apodoulou	Pig	0.709083
3331	Armenoi-500(37)	Armenoi	Human	0.709191
3529	ARM-501	Armenoi	Human	0.709114
3333	ARM-501(38)	Armenoi	Human	0.709415
3335	ARM-502(39)	Armenoi	Human	0.709122
3337	ARM-503 Tomb 160 (40)	Armenoi	Human	0.709032
3339	ARM-504 Tomb 200 (41)	Armenoi	Human	0.709228
3531	ARM-521	Armenoi	Human	0.709226
3533	ARM-522	Armenoi	Human	0.709153
3524	ARM-524	Armenoi	Human	0.708741
3288	Chamalevri-22 (64)	Chamalevri	Sheep/Goat	0.708560
3270	Chamalevri-5 (47)	Chamalevri	Sheep/Goat	0.708774
3278	Chamalevri-15 (57)	Chamalevri	Sheep/Goat	0.708782
3277	Chamalevri-14 (56)	Chamalevri	Sheep/Goat	0.708786
3279	Chamalevri-16 (58)	Chamalevri	Sheep/Goat	0.708789
3285	Chamalevri-26 (68)	Chamalevri	Sheep/Goat	0.708849
3281	Chamalevri-19 (61)	Chamalevri	Sheep/Goat	0.708869
3296	Chamalevri-30 (72)	Chamalevri	Sheep/Goat	0.708996
3266	Chamalevri-12 (54)	Chamalevri	Sheep/Goat	0.709080
3286	Chamalevri-27 (69)	Chamalevri	Cattle	0.708609
3290	Chamalevri-25 (67)	Chamalevri	Cattle	0.708853
3258	Chamalevri-3 (45)	Chamalevri	Cattle	0.708868
3295	Chamalevri-28 (70)	Chamalevri	Cattle	0.708926
3284	Chamalevri-24 (66)	Chamalevri	Cattle	0.708950
3292	Chamalevri-29 (71)	Chamalevri	Cattle	0.709006
3268	Chamalevri-13 (55)	Chamalevri	Pig	0.709044
3264	Chamalevri-10 (52)	Chamalevri	Pig	0.709048
3260	Chamalevri-4 (46)	Chamalevri	Pig	0.709030
3256	Chamalevri-I (43)	Chamalevri	Pig	0.708771
3275	Chamalevri-11 (53)	Chamalevri	Pig	0.709099
3273	Chamalevri-7 (49)	Chamalevri	Pig	0.709136
3262	Chamalevri-9 (51)	Chamalevri	Fallow Deer	0.709038
3283	Chamalevri-18 (60)	Chamalevri	Dog	0.709039
3293	Chamalevri-21 (63)	Chamalevri	Equid	0.709077
3271	Chamalevri-6 (48)	Chamalevri	Equid	0.709101
4549	Chania-1 T.27	Chania	Human	0.709094
4550	Chania-2 T.27	Chania	Human	0.709098
4554	Chania-5 Tomb 12	Chania	Human	0.708912
4556	Chania-6 Tomb 12	Chania	Human	0.709023
4557	Chania-7	Chania	Human	0.709010
4559	Chania-9 Tomb 13	Chania	Human	0.709085
4561	Chania-11 Tomb 13	Chania	Human	0.709033
4563	Chania-12 Tomb 18	Chania	Human	0.709075
4565	Chania-14 Tomb 18	Chania	Human	0.709039
4566	Chania-15 Tomb 39	Chania	Human	0.708329
4568	Chania-17 Tomb 35	Chania	Human	0.708825
4570	Chania-19 Tomb 37	Chania	Human	0.709009
3326	Gerani 1	Gerani	Human	0.709091
3329	Gerani 2	Gerani	Human	0.709065
4536	Kastelli-17	Kastelli	Sheep/Goat	0.708795
4514	Kastelli-2	Kastelli	Sheep/Goat	0.708859
4539	Kastelli-19	Kastelli	Sheep/Goat	0.708892
4527	Kastelli-11	Kastelli	Sheep/Goat	0.709054
4526	Kastelli-10	Kastelli	Sheep/Goat	0.709055
4515	Kastelli-3	Kastelli	Sheep/Goat	0.709260
4522	Kastelli-8	Kastelli	Pig	0.709095
4531	Kastelli-14	Kastelli	Pig	0.708986
4529	Kastelli-12	Kastelli	Pig	0.709012
4541	Kastelli-20	Kastelli	Pig	0.709035
4534	Kastelli-16	Kastelli	Pig	0.709057
4537	Kastelli-18	Kastelli	Cattle	0.709092
4520	Kastelli-7	Kastelli	Fallow deer	0.709114
4512	Kastelli-1	Kastelli	dog	0.709035
4543	Kastelli-22	Kastelli	Equid	0.708925
3317	Maroulas- 2(74)	Maroulas	Human	0.709121
3318	Maroulas-4 (76)	Maroulas	Human	0.709131
3319	Maroulas-6 (78)	Maroulas	Human	0.709122
3299	Melidoni-2 (12)	Melidoni	Human	0.708464
3307	Melidoni-9 (19)	Melidoni	Pig	0.708415
3298	Melidoni-1 (11)	Melidoni	Pig	0.708979
3305	Melidoni-8 (18)	Melidoni	Cattle	0.708812
3304	Melidoni-5 (15)	Melidoni	Cattle	0.708867
3308	Melidoni-10 (20)	Melidoni	Sheep/Goat	0.708958
4614	Mochlos-27	Mochlos	Human	0.708780
4616	Mochlos-28	Mochlos	Human	0.708995
4618	Mochlos-29	Mochlos	Human	0.708801
4620	Mochlos-30	Mochlos	Human	0.709006
4622	Mochlos-31	Mochlos	Human	0.708795
4624	Mochlos-32	Mochlos	Human	0.708907
4626	Mochlos-33	Mochlos	Human	0.708960
4655	Mochlos-22	Mochlos	Human	0.708874
4657	Mochlos-23	Mochlos	Human	0.708912
4659	Mochlos-24	Mochlos	Human	0.708723
4661	Mochlos-25	Mochlos	Human	0.708665
4663	Mochlos-26	Mochlos	Human	0.708825
4644	Mochlos-15	Mochlos	Sheep/Goat	0.708371
4634	Mochlos-7	Mochlos	Sheep/Goat	0.708448
4646	Mochlos-16	Mochlos	Sheep/Goat	0.708457
4642	Mochlos-14	Mochlos	Sheep/Goat	0.708478
4650	Mochlos-18	Mochlos	Pig	0.708546
4648	Mochlos-17	Mochlos	Pig	0.708829
4573	Nerokourou-2	Nerokourou	Sheep/Goat	0.708896
4574	Nerokourou-3	Nerokourou	Sheep/Goat	0.709196
4572	Nerokourou-1	Nerokourou	Sheep/Goat	0.709082
4575	Nerokourou-4	Nerokourou	Pig	0.708900
4576	Nerokourou-5	Nerokourou	Pig	0.708907
4577	Nerokourou-6	Nerokourou	Pig	0.709201
4498	PK-24	Palaikastro	Sheep/Goat	0.708651
4494	PK-22	Palaikastro	Sheep/Goat	0.708939
4464	PK-1	Palaikastro	Sheep/Goat	0.709057
4481	PK-13	Palaikastro	Sheep	0.709140
4474	PK-8	Palaikastro	Cattle	0.708711
4492	PK-21	Palaikastro	Cattle	0.708852
4476	PK-10	Palaikastro	Cattle	0.708856
4502	PK-27	Palaikastro	Cattle	0.709011
4506	PK-29	Palaikastro	Pig	0.709071
4484	PK-15	Palaikastro	Pig	0.709136
4504	PK-28	Palaikastro	Pig	0.709110
4471	PK-6	Palaikastro	Dog	0.709077
4490	PK-20	Palaikastro	Dog	0.709111
4479	PK-12	Palaikastro	Dog	0.709126
4546	Perama-3	Perama	Human	0.709023
4547	Perama-4	Perama	Human	0.709010
4548	Perama-5	Perama	Human	0.709085
3352	Platyvola-2	Platyvola	Human	0.709137
3353	Platyvola-3	Platyvola	Human	0.709160
3355	Platyvola-5	Platyvola	Human	0.709131
3357	Platyvola-6	Platyvola	Human	0.709137
3359	Platyvola-10	Platyvola	Human	0.709019
3361	Platyvola-14	Platyvola	Human	0.709093
4605	Pseira-2	Pseira	Sheep/Goat	0.708655
4606	Pseira-3	Pseira	Cattle	0.708835
1253	Vrysinas-9 (87)	Vrysinas	Pig	0.708649
3320	Vrysinas-1(79)	Vrysinas	Pig	0.708697
3324	Vrysinas-10(88)	Vrysinas	Pig	0.708741
3323	Vrysinas-6(84)	Vrysinas	Sheep/Goat	0.708870
3321	Vrysinas-2(80)	Vrysinas	Sheep/Goat	0.709006
3325	Vrysinas-12 (93)	Vrysinas	Fallow deer	0.708877
3313	Zoniana-5 (5)	Zoniana	Sheep/Goat	0.708230
3310	Zoniana-1 (1)	Zoniana	Sheep/Goat	0.708711
3312	Zoniana-2 (2)	Zoniana	Pig	0.708470
1238	Zoniana-10 (10)	Zoniana	Cattle	0.708732
3315	Zoniana-8 (8)	Zoniana	Cattle	0.708845
3314	Zoniana-7 (7)	Zoniana	Cattle	0.709282

### DNA analysis

We processed 16 samples from 12 individuals from the sites Armenoi and Gerani for DNA analyses (Table 6). Pre-PCR steps took place in the clean room facilities of the Institute for Archaeological Sciences at the University of Tübingen, Germany. After surface irradiation with ultraviolet light, the tooth was sawn apart transversally at the border of crown and root, and dentine powder from the inside the crown was sampled using a sterile dentistry drill. Bone surface at the sample area was removed with a dental drill, before extracting bone powder using a new drill bit. DNA extraction was carried out on around 100 mg of dentine powder per sample according to Dabney et al. [50]. DNA libraries were prepared from 10 or 20μl of extract each [51, 52], and enriched for human mitochondrial DNA using a bead-based hybridization protocol [53]. Negative controls were included in the extraction and library preparation and taken along for all further processing steps.

**Table 6:** mtDNA results (haplogroup, coverage, contamination estimation) from 12 Bronze Age humans from the site of Armenoi, and two Neolithic humans from the site of Gerani.

Sample ID	Tomb	Skeletal element	Shotgun data processing			mtDNA capture data processing			
			Unique mapped human reads	Endogenous DNA (%)	Damage 1st Base 5’	Mean Coverage	Damage 1st Base 5’	Contamination estimate	Haplogroup assignment
Gerani 1	n/a	bone	20	0.013	0.00	0.45	0.11	n/a	n/a
Gerani 1	n/a	tooth	33	0.032	0.56	28.45	0.48	0.01–0.03	T2b2b
Gerani 2	n/a	bone	35	0.083	0.15	0.30	0.33	n/a	n/a
Gerani 2	n/a	tooth	104	0.071	0.50	21.43	0.43	0–0.02	H
T146 Armenoi	T146	bone	41	0.049	0.18	0.37	0.13	n/a	n/a
T146 A Armenoi	T146	bone	105	0.089	0.08	0.41	0.05	n/a	n/a
T146 A Armenoi	T146	tooth	32	0.084	0.08	2.83	0.49	0–0.02	H84
S-EVA 1263, Armenoi 503	89I	tooth	365	0.357	0.57	374.51	0.45	0.01–0.03	U5a1e
S-EVA 1264, Armenoi 504	89B	tooth	228	0.391	0.37	125.03	0.42	0.01–0.03	HV
S-EVA 3521, Armenoi 522	89A	tooth	31	0.048	0.25	0.64	0.45	n/a	n/a
Sample 1 Armenoi	230	tooth	56	0.031	0.13	8.89	0.53	0.01–0.03	H59
3A Armenoi	230	tooth	12	0.014	0.00	26.51	0.55	0.01–0.03	H59
3B Armenoi	230	tooth	10	0.011	0.00	53.12	0.52	0.01–0.03	H59
A Armenoi	230	tooth	83	0.043	0.21	14.26	0.52	0.01–0.03	H59
A Armenoi	230	tooth	35	0.03	0.00	0.14	0.33	n/a	n/a
S-EVA 3529, Armenoi 501	55G	tooth	521	0.184	0.56	25.25	0.51	0.01–0.03	T2b25

Sequencing of shotgun libraries and mitochondrial-DNA-enriched libraries took place at the facilities of the Frauenklinik of the University of Tübingen on an Illumina HiSeq 2500 for 2 × 101 + 8 cycles.

Base call files produced by the instrument’s software were converted to raw sequences that were demultiplexed according to the index combinations they received during library preparation. The software pipeline EAGER [54] was used to clip adapter sequences, map reads to the reference—hg19 for shotgun data and Reconstructed Sapiens reference sequence (RSRS) for mitochondrial capture data—using Burrows–Wheeler Aligner (BWA) [55] with parameters “-l 10000 -n 0.01,” and to remove duplicate reads. Mitochondrial consensus sequences were called while jointly estimating the rate of deamination damage and contamination from present-day human sources with the probabilistic iterative method applied in the software schmutzi [56] using the accompanying tool log2fasta with parameter -q 20. Poly-C regions and mutational hotspots at positions 303–315, 515–522, and 16519 were masked. Sex was assigned on the basis of the shotgun-sequencing data [57] and results are reported S1 Table, but we caution that these are extremely uncertain due to the low number of mapping shotgun reads. Mitochondrial haplogroups were assigned manually by consulting the output of Haplofind [58] and Haplogrep2.0 [59].

## Supporting information

S1 Table Details of the DNA extractions. (XLSX)

S1 File (DOCX)
